# Dual-Broadband Topological Photonic Crystal Edge State Based on Liquid Crystal Tunability

**DOI:** 10.3390/ma18122778

**Published:** 2025-06-12

**Authors:** Jinying Zhang, Bingnan Wang, Jiacheng Wang, Xinye Wang, Yexiaotong Zhang

**Affiliations:** 1 Beijing Key Lab for Precision Optoelectronic Measurement Instrument and Technology, School of Optics and Photonics, Beijing Institute of Technology, Beijing 100081, China; 3120215361@bit.edu.cn (B.W.); 3120225346@bit.edu.cn (J.W.); 3120205353@bit.edu.cn (X.W.); 3120220661@bit.edu.cn (Y.Z.); 2 Yangtze Delta Region Academy of Beijing Institute of Technology, Jiaxing 314001, China; 3 National Key Laboratory on Near-Surface Detection, Beijing 100072, China

**Keywords:** liquid crystal, valley photonic crystal, dual band, edge states, birefringence, terahertz

## Abstract

The rapid advancements in optical communication and sensing technologies have significantly increased the demand for advanced tunable spectral systems. This study presents a dual-band terahertz transmission and manipulation approach by leveraging the topologically protected properties of valley-topological photonic crystal edge states. The designed structure facilitates the excitation of the K valley within the range of 0.851–0.934 THz and the K′ valley from 1.604 to 1.686 THz, while also demonstrating anomalous refraction and birefringence. The calculated emission angles, derived through momentum matching, enable transitions between single-wave and dual-wave emissions and allow for precise angle control. The introduction of the liquid crystal material NJU-LDn-4 enables continuous tuning of the dual-band spectral range under a varying electric field, broadening the operating frequency bands to the ranges of 0.757–0.996 THz and 1.426–1.798 THz, respectively. These findings suggest promising applications in tunable filter design, optical communication, photonic computing, optical sensing, and high-resolution imaging, particularly in novel optical devices requiring precise control over spectral characteristics and light propagation.

## 1. Introduction

Spectral tunable technology enables the dynamic adjustment of light wavelengths or frequencies and holds significant potential for applications in a wide range of fields, including optical communication [1], lidar [2], infrared detection [3], photovoltaic solar energy [4,5], medical imaging [6], quantum computing [7,8], telecommunications [9,10,11], and sensing technologies [12,13]. Existing implementations of spectral tunable technologies include filter arrays [14,15], acousto-optic tuning devices [16,17], liquid crystal tunable filters [18,19,20], and various photonic crystal metamaterials [21,22,23,24,25]. In 2010, J.E. Antonio-Lopez [26] introduced a tunable multimode interference (MMI) filter composed of a multimode fiber (MMF) spliced with two single-mode fibers (SMFs). The peak wavelength of this filter exhibits a linear relationship with the length of the MMF. By utilizing a capillary tube filled with a liquid of matching refractive index, the effective MMF length can be extended, allowing for wavelength tuning. This MMI-based tunable optical filter offers advantages such as a simple structure, low cost, high stability, and ease of operation. However, its tuning range is constrained by the physical length of the capillary tube and the intrinsic properties of the MMF. Although a tuning range of 30 nm is achievable, the bandwidth remains narrow, and the wavelength precision is limited due to liquid-based modulation. Consequently, the robustness of this method is generally inferior to that of other advanced tunable filtering techniques. Ligang Huang et al. [27] proposed a broadband acousto-optic tunable fiber laser employing dual semiconductor optical amplifiers (SOAs) within a ring cavity structure. The configuration integrates an acousto-optic tunable filter and two SOAs with distinct gain characteristics to enable broad spectral tunability. This design provides an extended tuning range of 120 nm (1507–1627 nm), offering a faster tuning speed and improved linearity, and demonstrating potential applications in high-capacity optical communication, distributed fiber sensing, and gas detection. However, despite structural optimization aimed at minimizing insertion loss, a considerable level of insertion loss persists, negatively impacting the overall efficiency of the laser. Additionally, although the architecture is simpler than that of conventional grating-based lasers, it incorporates several fiber components—such as acousto-optic filters, SOAs, and optical couplers—and the tuning range becomes fixed once configured. The associated cost also remains relatively high. Therefore, there is a critical need to develop a spectral tuning method that offers a simpler structure, high robustness, and the flexibility to support adjustable tuning ranges, including dual-band or multi-band capabilities.

Previous investigations have investigated the influence of liquid crystals on the band structures of valley-topological photonic crystals [28,29] and explored the phenomenon of anomalous refraction [30,31]. However, these studies have primarily focused on single-band operation, lacking a comprehensive analysis of the underlying mechanisms that contribute to anomalous refraction. In our previous work, we designed a novel two-dimensional valley-topological photonic crystal [22]. This valley-topological photonic crystal exhibits edge states in two separate bands, and we verified that these edge states demonstrate remarkable robustness against impurities, defects, and sharp corners. This photonic crystal enables independent excitation of valley states at the K and K′ points through excitation by a chiral source [23], facilitating simultaneous valley excitation over specific frequency ranges. Nevertheless, due to inherent structural constraints, this excitation requires the chiral source to be placed internally, thereby presenting significant challenges for experimental implementation. To address this challenge, we redesigned the system architecture to develop a liquid crystal tunable, dual-broadband topological photonic crystal edge state, thereby successfully achieving both anomalous refraction and birefringence. By employing the liquid crystal material NJU-LDn-4 [32,33,34,35,36], we achieved systematic tunability of the dual-band spectrum under a continuously varying electric field, thereby enhancing filtering performance and expanding the information transmission capacity. The resulting emission angles are shown to be independent of the incidence angle, highlighting the substantial application potential of this approach in terahertz filtering, optical sensing, communication [37], switching, and photonic computing.

## 2. Materials and Methods

### 2.1. Dual-Broadband Valley-Topological Photonic Crystal

**Figure 1:**
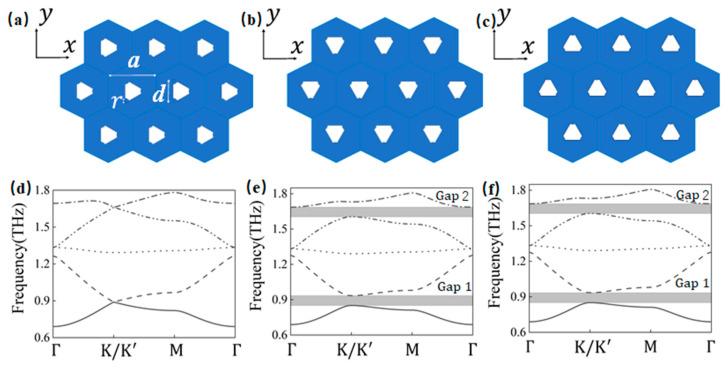
Schematic of the unit cell structure of the two-dimensional topological photonic crystal and its band structure. (**a**): Schematic of the unit cell structure at θ=0° , which exhibits *C*_3*v*_ symmetry; (**b**): schematic of the unit cell structure at θ=−30° , where *C*_3*v*_ symmetry is broken; (**c**): schematic of the unit cell structure at θ=30° ; (**d**): band structure of the unit cell at θ=0° , exhibiting double Dirac cone protection; (**e**): band structure of the unit cell at θ=−30° , where the band gap is maximized at this rotation angle; (**f**): band structure of the unit cell structure at θ=30° .

In our previous study [22], a unit cell structure of a two-dimensional topological photonic crystal with *C*_3*v*_ symmetry was constructed, as illustrated in Figure 1. The unit cell comprises triangular units with arc-shaped cuts, where the white regions indicate metallic scatterers and the blue regions represent a dielectric material with a refractive index of $n_{1}=1.6$ . The structural parameters are defined as follows: the unit cell is hexagonal, with a lattice constant of $a=\;100\sqrt{3\;}\mu m$ . Each scatterer is shaped as an equilateral triangle with a side length of $d=\frac{3\sqrt{3}a}{10}$ , from which a circular sector with a radius of r=0.1 d is removed from each vertex. The scatterers are rotatable about their central axis, and the rotation angle is denoted by θ ; clockwise rotation is defined as positive, while counterclockwise rotation is defined as negative.

As shown in Figure 1a,d, when θ=0° , the band structure of the unit cell exhibits the characteristics of double Dirac cone protection. However, when θ is non-zero, the spatial inversion symmetry is broken, leading to the loss of Dirac cone protection. Figure 1b,c illustrate that at θ=−30° and θ=30° , the double Dirac cones open two distinct band gaps, which we designate as Gap 1 and Gap 2. The band gap at θ=−30° is shown in Figure 1e, while the band gap at θ=30° is depicted in Figure 1f. The propagation of frequencies within the band gaps is completely inhibited in the array composed of unit cells. The frequency ranges corresponding to Gap 1 and Gap 2 are 0.851–0.934 THz and 1.604–1.686 THz, respectively.

The Chern number of the band gap is calculated using the k.p perturbation method [38], according to the following expression:

(1) $$C_{K/K\prime }=\frac{1}{2\pi }\int_{HBZ}\;\Omega_{n}\left(k\right)d^{2}k$$

where HBZ denotes the half Brillouin zone and $\Omega_{n}\left(k\right)$ represents the Berry curvature, given by the following expression:

(2) $$\Omega_{n}\left(k\right)=\nabla_{k}\times A(k)$$

Here, n indicates the band index and $A_{n}(k)$ is the Berry connection. By calculating the Berry curvature, the Chern number can be obtained, reflecting the topological characteristics of the material. By assembling the photonic crystal composed of unit cells with $\left|\Delta C\right|=1$ , topologically protected edge states will exist at the boundaries. These edge states exhibit strong robustness against defects and impurities, thereby improving the practical viability of these crystals for optical applications.

### 2.2. Excitation of the Edge States

**Figure 2:**
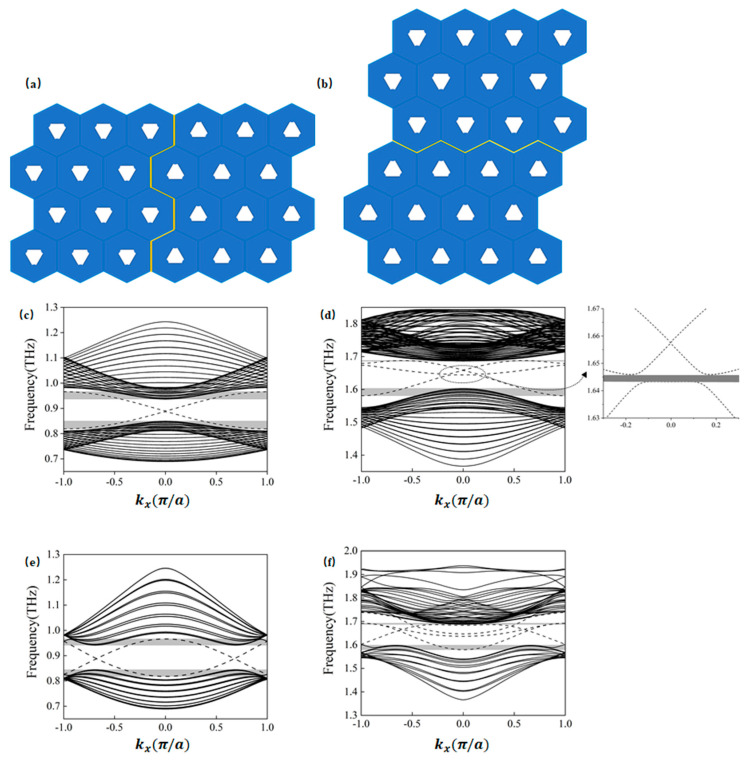
Schematic of armchair and zigzag edge states in the topologically protected two-dimensional photonic crystal and their band structures. The yellow regions denote the boundaries, with dashed lines representing the edge states and solid lines indicating the bulk states. The shaded areas depict the overlapping frequency regions of edge states and bulk states. (**a**) Armchair edge states; (**b**) zigzag edge states; (**c**) band structure near Gap 1 for armchair edge states; (**d**) band structure near Gap 2 for armchair edge states, where the enlarged region highlights the band gap; (**e**) band structure near Gap 1 for zigzag edge states; (**f**) band structure near Gap 2 for zigzag edge states.

Edge states can be categorized into armchair and zigzag types, depending on their geometric terminations, as illustrated in Figure 2a,b. A detailed description of the zigzag and armchair edge states, along with an analysis of their excitation mechanisms, is presented in [22]. Figure 2c,f show the band structures of these boundaries within the vicinity of Gap 1 and Gap 2. The dashed lines represent edge states formed by supercell structures, while the solid lines denote bulk states. In the band structure of the armchair type edge state shown in Figure 2d, a narrow band gap emerges within the frequency range of 1.643–1.646 THz. The shaded areas indicate the frequency regions where edge and bulk states coexist. Although edge states may arise within these overlapping regions, the electromagnetic energy is not strictly confined to the boundary. In applications such as long-distance light transmission, optical communication systems, and photonic computing [39], excessive energy leakage is unacceptable.

**Figure 3:**
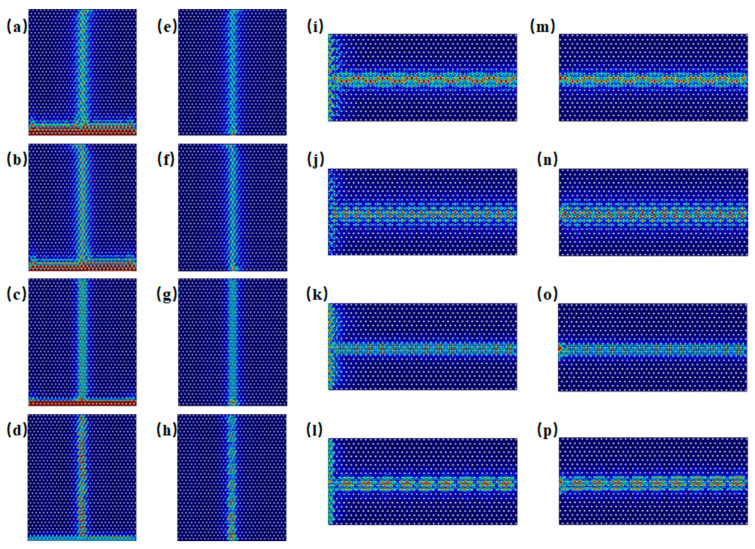
Excitation of armchair and zigzag edge states at selected frequencies. (**a**–**d**) Excitation at 0.860 THz, 0.93 THz, 1.61 THz, and 1.68 THz along the entire side of the structure, showing boundary-confined energy propagation in the armchair configuration. (**e**–**h**) Excitation into the armchair boundary at 0.86 THz, 0.93 THz, 1.61 THz, and 1.68 THz, respectively. (**i**–**l**) Excitation at 0.860 THz, 0.93 THz, 1.61 THz, and 1.68 THz along the entire side of the zigzag type boundary, respectively. (**m**–**p**) Excitation into the zigzag boundary at 0.86 THz, 0.93 THz, 1.61 THz, and 1.68 THz, respectively.

Edge states of both armchair and zigzag configurations are excited within the frequency ranges of Gap 1 (0.851–0.934 THz) and Gap 2 (1.604–1.686 THz). Figure 3 shows the excitation of edge states for both configurations at incident frequencies of 0.86 THz, 0.93 THz, 1.61 THz, and 1.68 THz. For computational efficiency, the metallic scatterers were modeled as lossless perfect electric conductors (PECs), and material dielectric losses were excluded (see Supplementary Materials Note S1 for implementation details). It is observed that excitation across the entire side of the structure tends to preferentially activate bulk states, whereas boundary-localized excitation more effectively generates topologically protected edge states. For example, in Figure 3a at 0.86 THz, even though excitation occurs along the entire side of the structure, it is noticeable that energy, although incident along the entire side, can propagate only through the boundary. When waves at frequencies within Gap 1 and Gap 2 are incident, the energy is theoretically confined to the edge region without dissipation, demonstrating ideal waveguide characteristics under lossless conditions.

**Figure 4:**
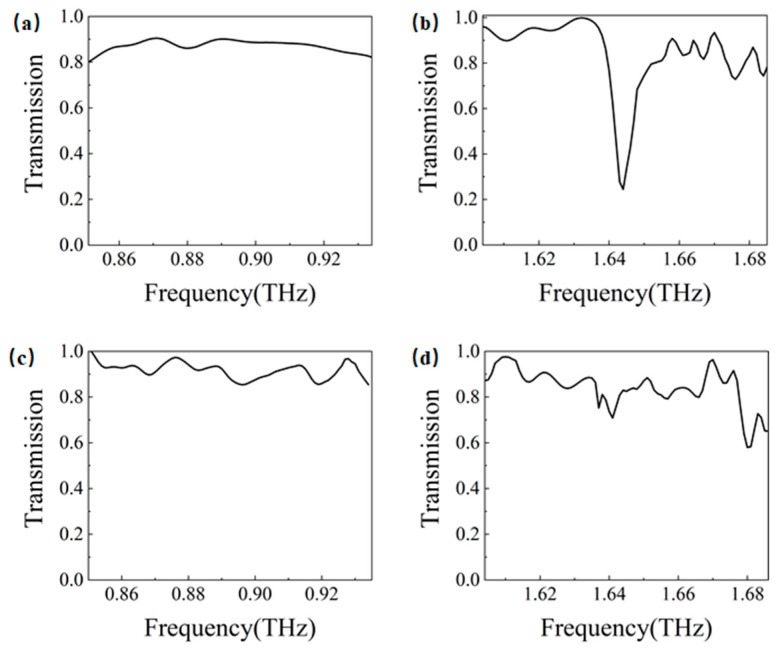
Transmission at the Gap 1 and Gap 2 boundaries for armchair and zigzag edge states: (**a**) the transmission of armchair edge states at Gap 1; (**b**) the transmission of armchair edge states at Gap 2; (**c**) the transmission of zigzag edge states at Gap 1; (**d**) the transmission of zigzag edge states at Gap 2.

However, realistic materials exhibit intrinsic losses. To account for the loss characteristics of both metallic and dielectric components, we employed the actual material parameters of silver [40] for the metal layer and CS_2_ [41] for the dielectric layer in our calculations of transmission at the Gap 1 and Gap 2 boundaries for armchair and zigzag edge states, respectively. The results are presented in Figure 4. Notably, the armchair edge state at the Gap 2 boundary shows a pronounced transmission dip near 1.644 THz, which directly corresponds to the band gap identified in the band structure analysis discussed earlier (see Supplementary Materials Note S2 for implementation details).

**Figure 5:**
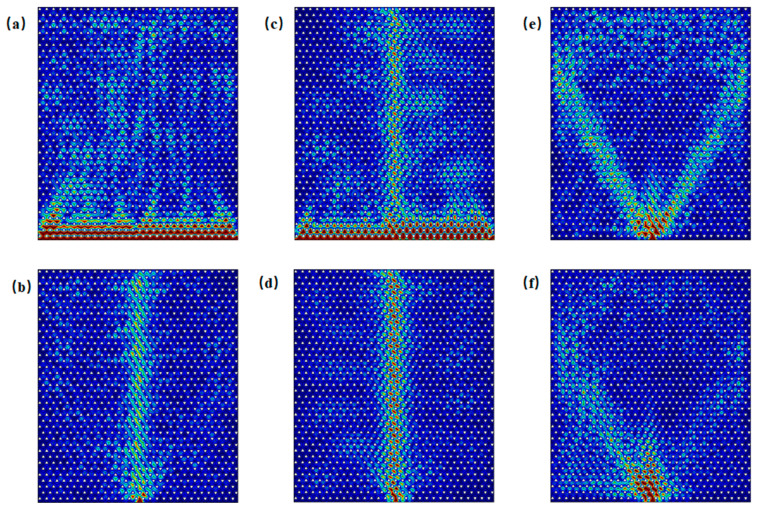
Schematic of excitations of armchair edge states near Gap 1: (**a**) energy distribution under excitation at 0.830 THz incident along the entire side; (**b**) energy distribution under excitation at 0.83 THz incident into the boundary; (**c**) energy distribution under excitation at 0.95 THz incident along the side; (**d**) energy distribution under excitation at 0.95 THz incident into the boundary; (**e**) energy distribution under excitation at 0.818 THz incident into the boundary; (**f**) energy distribution under excitation at 0.967 THz incident into the boundary.

As illustrated in the band structure shown in Figure 2c, both bulk states and edge states coexist in the armchair boundary configuration near Gap 1, specifically in the frequency ranges of 0.819–0.850 THz and 0.937–0.966 THz. As an example, when the frequency is 0.830 THz, Figure 5a displays the scenario in which energy is incident along the entire side of the structure, allowing for the simultaneous excitation of both edge states and bulk states. In contrast, Figure 5b shows the case where excitation occurs only at the boundary. Similarly, when the frequency is 0.950 THz, Figure 5c depicts the situation where energy is again incident along the entire side of the structure, resulting in the simultaneous excitation of edge and bulk states. Figure 5d, on the other hand, shows the scenario where excitation is localized only at the boundary. When the frequency is below 0.819 THz or above 0.966 THz, as indicated by the cases at 0.818 THz in Figure 5e and 0.967 THz in Figure 5f, edge states do not exist, and waves incident at the boundary cannot propagate through it.

**Figure 6:**
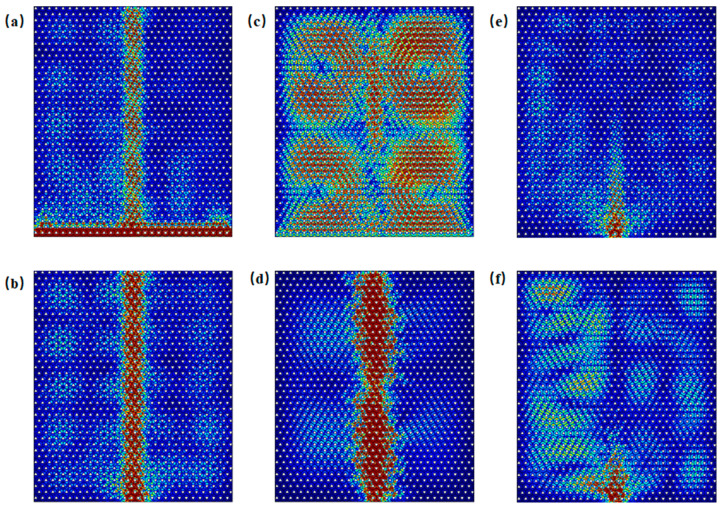
Schematic of excitations of armchair edge states near Gap 2: (**a**) energy distribution under excitation at 1.590 THz incident along the entire side; (**b**) energy distribution under excitation at 1.590 THz incident into the boundary; (**c**) energy distribution under excitation at 1.6870 THz incident along the side; (**d**) energy distribution under excitation at 1.687 THz incident into the boundary; (**e**) energy distribution under excitation at 1.580 THz incident into the boundary; (**f**) energy distribution under excitation at 1.690 THz incident into the boundary.

As shown in the band structure of Figure 2d, in proximity to Gap 2 in the armchair structure, edge states and bulk states are observed to coexist within the frequency ranges of 1.581–1.603 THz and 1.686–1.688 THz. In Figure 6a, at a frequency of 1.590 THz, under excitation along the side, both edge and bulk states are simultaneously excited. Figure 6b illustrates excitation at 1.590 THz with waves incident into the boundary. As shown in Figure 6c, at a frequency of 1.687 THz, under excitation along the side, both edge and bulk states are again excited. Figure 6d demonstrates excitation at 1.687 THz with waves incident into the boundary. When the frequency is below 1.581 THz or above 1.688 THz, edge states are absent. Figure 6e,f illustrate excitation at 1.580 THz and 1.690 THz with waves incident into the boundary, in which only bulk states are excited.

**Figure 7:**
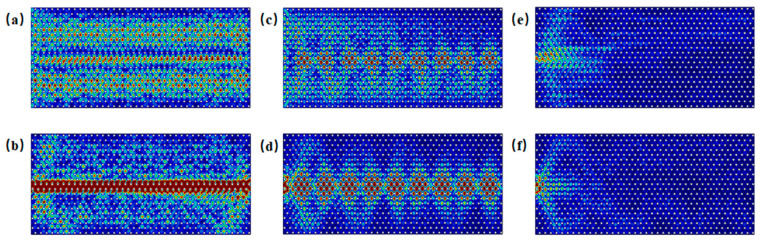
Schematic illustration of zigzag edge state excitations near Gap 1: (**a**) energy distribution under excitation at 0.825 THz incident along the side; (**b**) energy distribution under excitation at 0.825 THz incident into the boundary; (**c**) energy distribution under excitation at 0.955 THz incident along the side; (**d**) energy distribution under excitation at 0.955 THz incident into the boundary; (**e**) energy distribution under excitation at 0.817 THz incident into the boundary; (**f**) energy distribution under excitation at 0.967 THz incident into the boundary.

As shown in the band structure in Figure 2e, zigzag edge states are present near the Gap 1 region, where edge and bulk states coexist within the frequency ranges of 0.818–0.844 THz and 0.942–0.966 THz. Figure 7a displays the energy distribution under excitation at 0.825 THz from the entire side, while Figure 7b shows the distribution under excitation from the boundary at the same frequency. Figure 7c illustrates the energy distribution under excitation at 0.955 THz from the entire side, and Figure 7d depicts the response under excitation from the boundary, indicating the simultaneous excitation of edge and bulk states. When the frequency is below 0.818 THz or above 0.966 THz, as seen in Figure 7e at 0.817 THz and in Figure 7f at 0.967 THz, edge states are absent; only bulk states are excited under boundary excitation.

**Figure 8:**
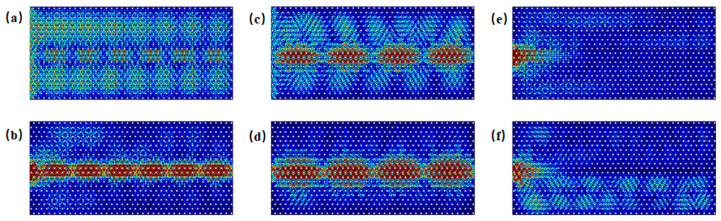
Schematic of excitations of zigzag edge states near Gap 2: (**a**) energy distribution under excitation at 1.587 THz incident along the side; (**b**) energy distribution under excitation at 1.587 THz incident into the boundary; (**c**) energy distribution under excitation at 1.690 THz incident along the side; (**d**) energy distribution under excitation at 1.690 THz incident into the boundary; (**e**) energy distribution under excitation at 1.578 THz incident into the boundary; (**f**) energy distribution under excitation at 1.694 THz incident into the boundary.

As shown in the band structure in Figure 2f, zigzag edge states are present near the Gap 2 region, where edge and bulk states coexist within the frequency ranges of 1.580–1.598 THz and 1.688–1.692 THz. Figure 8a illustrates the energy distribution under excitation at 1.587 THz incident along the side, while Figure 8b shows the distribution under excitation at 1.587 THz incident into the boundary. Figure 8c depicts the energy distribution under excitation at 1.690 THz incident along the side, and Figure 8d presents the corresponding distribution under excitation into the boundary. In all cases, both edge and bulk states are simultaneously excited. When the frequency is below 1.580 THz or above 1.692 THz, edge states are absent under boundary excitation, as seen in Figure 8e at 1.578 THz and in Figure 8f at 1.694 THz; only bulk states are excited in these cases.

In analyzing edge state band structures for armchair and zigzag configurations, although the edge state ranges shown in the band diagrams extend beyond Gap 1 and Gap 2, it is critical to identify the frequency ranges where edge and bulk states coexist, and to exclude those that could degrade waveguide transmission performance. This filtering process is key to maintaining effective and robust waveguide operation. As shown in Figure 1, when the spatial inversion symmetry of the unit cell structure is broken, the Dirac cone degeneracy in the energy bands is lifted. This phenomenon reveals the opening of bulk state bands, corresponding to the conditions discussed in this section, where only edge states exist in the absence of bulk states. To maintain robust energy transmission during edge state excitation, the following analysis will therefore emphasize the operational conditions under which only edge states are present, specifically within Gap 1 and Gap 2.

## 3. Anomalous Refraction, Birefringence, and Momentum Matching Conditions of Valley Edge State Excitation

### 3.1. Anomalous Refraction and Birefringence of Valley Edge States

**Figure 9:**
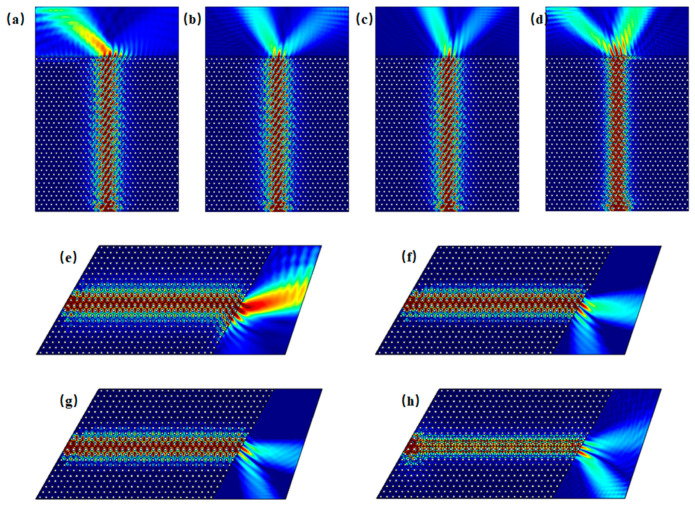
Anomalous refraction and birefringence behavior in dual-band valley-topological photonic crystals under varying refractive index conditions: (**a**) when $n_{2}=1.0$ , the armchair edge state exhibits anomalous refraction characteristics at frequencies in Gap 1; (**b**) when $n_{2}=1.6$ , the armchair edge state shows birefringence characteristics at frequencies in Gap 1; (**c**) when $n_{2}=2.0$ , as the refractive index of the surrounding environment increases, the armchair edge state experiences a reduction in the refractive angles of birefringence in Gap 1, with the two exiting beams gradually converging; (**d**) when $n_{2}=1.0$ , the armchair edge state demonstrates birefringence characteristics at frequencies in Gap 2, although the distribution of birefringence is symmetric compared to Gap 1; (**e**–**h**) depict the anomalous refraction and birefringence characteristics of zigzag edge states, with analysis methods similar to those of the armchair type.

The electric field distribution of edge states when exiting into the surrounding environment is illustrated in Figure 9. Taking $n_{1}=1.6$ with frequencies at 0.880 THz in Gap 1 and 1.610 THz in Gap 2 as an example, armchair edge states are excited from below, while zigzag edge states are excited from the left, with $n_{2}$ representing the refractive index of the surrounding environment. Figure 9a–c depict the anomalous refraction and birefringence of the edge states at 0.88 THz under armchair boundary conditions for $n_{2}=1.0$ , 1.6 , and 2.0 , respectively. Figure 9d shows the birefringence of the edge state at 1.610 THz under $n_{2}=1.0$ . Figure 9e–g illustrate the same effects under zigzag boundary conditions for $n_{2}=1.0$ , 1.6 , and 2.0 at 0.880 THz, while Figure 9h presents the birefringence for the 1.610 THz edge state at $n_{2}=1.0$ . Regardless of whether under armchair or zigzag boundary conditions, in the frequency range at Gap 1, when the value of $n_{2}$ is relatively low, the outgoing light exhibits characteristics of anomalous refraction. As $n_{2}$ gradually increases, the outgoing light transitions from anomalous refraction to birefringence, with differing exit angles for the two beams. When the frequency is within the range of Gap 2, birefringence effects are observed regardless of the value of $n_{2}$ , although the distribution of birefringence exhibits symmetrical characteristics compared to Gap 1.

### 3.2. Momentum Matching Method for Anomalous Refraction and Birefringence of Valley Edge States

Whether in armchair or zigzag boundary configurations, the emitted light deviates from conventional laws of refraction, instead exhibiting a consistent angular relationship with the exit boundaries. This phenomenon suggests that light propagation is strongly influenced by the boundary geometry, highlighting the role of photonic topology beyond classical optical predictions. The momentum matching method is employed as a novel analytical approach to investigate the physical mechanism governing the angular dependence between the emitted light and the output boundary.

**Figure 10:**
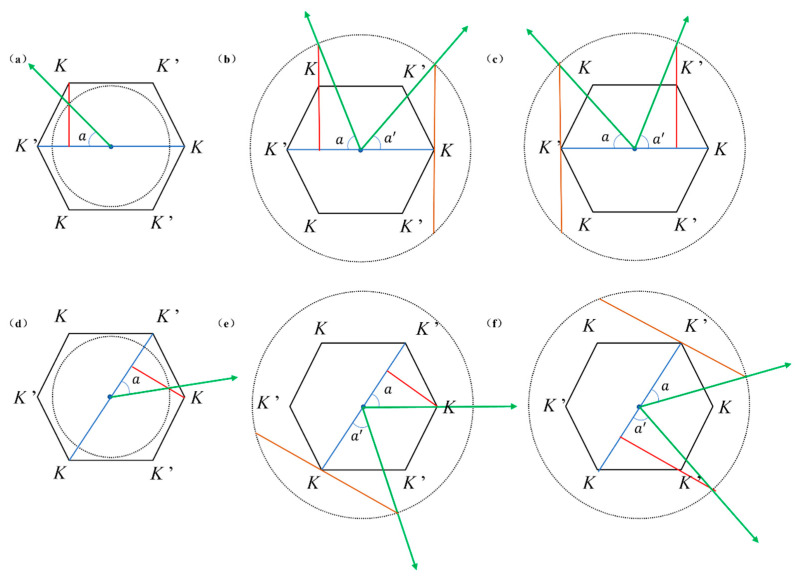
Momentum matching analysis of anomalous refraction and birefringence in armchair and zigzag valley edge states, the blue lines indicating the exit sides, and the green arrows representing the exit direction, the red line indicates the vertical line: (**a**) armchair edge states exhibiting anomalous refraction for excitation momentum below the K-valley momentum in Gap 1; (**b**) armchair edge states exhibiting birefringence for excitation momentum above the K-valley momentum in Gap 1; (**c**) birefringence of armchair edge states in Gap 2; (**d**) zigzag edge states exhibiting anomalous refraction for excitation momentum below the K-valley momentum in Gap 1; (**e**) zigzag edge states exhibiting birefringence for excitation momentum above the K-valley momentum in Gap 1; (**f**) birefringence of zigzag edge states in Gap 2.

The excitation of K valley edge states must satisfy the momentum matching condition. The K point is located at the vertices of the hexagon in the first Brillouin zone, as shown in Figure 10. The black boundary indicates the first Brillouin zone, with the black dashed lines representing the momentum of the excitation frequency, the blue lines indicating the exit sides, and the green arrows representing the exit direction. Thus, the momentum of the K valley is given by $\left|K\right|=\frac{2\pi }{a}\times \frac{2}{\sqrt{3}}\times \frac{1}{\sqrt{3}}=\frac{4\pi }{3a}=2.42\;\times \;10^{4}\;\left[1/m\right]$ . The excitation momentum is $\left|k\right|=\frac{2\pi f}{c/n_{2}}$ [30], where f is the excitation frequency and $n_{2}$ is the refractive index of the surrounding environment. The angle of anomalous refraction for the exiting light is given by $\cos\alpha =\frac{\left|K\right|}{2\left|k\right|}$ , while the exit angle α′ for the exiting light contributing to birefringence is defined as $\cos\alpha \prime =\frac{\left|K\right|}{\left|k\right|}$ . When $n_{1}=1.6$ and the frequency f is taken as 0.880 THz in Gap 1 and 1.610 THz in Gap 2, as illustrated in Figure 10a,b, for $f_{1}=0.880\;\mathrm{THz}$ with the surrounding environment $n_{2}=1$ (air), the computed value for $k_{1}$ is $1.84\;\times \;10^{4}\;[1/m]$ . The excitation momentum’s equal-frequency contour is enclosed by the first Brillouin zone. At this point, as shown in Figure 9a,e, the K valley is excited within the Gap 1 frequency band, with the exit angle $a_{1}$ relative to the exit boundary calculated as follows:

(3) $$a_{1}=\arccos\frac{2.42\times 10^{4}}{2\times 1.84\times 10^{4}}=48.88{}^{\circ}$$

When the surrounding medium is changed to $n_{2}=1.6$ , the computed value for $k_{2}=2.95\;\times \;10^{4}\;[1/m]$ . At this point, the equal-frequency contour of the excitation surrounds the first Brillouin zone, allowing for the simultaneous excitation of two different K valleys. The resulting angles are calculated as follows:

(4) $$a_{2}=\arccos\frac{2.42\times 10^{4}}{2\times 2.95\times 10^{4}}=65.78{}^{\circ},\;a_{2}\prime =\arccos\frac{2.42\times 10^{4}}{2.95\times 10^{4}}=34.88{}^{\circ}$$

The simulation results for the armchair and zigzag edge states are shown in Figure 9b and 9f, respectively, and the results calculated using the momentum matching method align with the simulation results. Increasing $n_{2}$ to 2.0 results in a continued increase in the exit angles, with $k_{3}$ computed as $k_{3}=3.69\;\times \;10^{4}\;[1/m]$ . The angles are calculated as follows:

(5) $$a_{3}=\arccos\frac{2.42\times 10^{4}}{2\times 3.69\times 10^{4}}=70.86{}^{\circ},\;a_{3}\prime =\arccos\frac{2.42\times 10^{4}}{3.69\times 10^{4}}=49.02{}^{\circ}$$

These computed results correspond to the simulation findings in Figure 9c,g for the armchair and zigzag edge states. When $f_{2}=1.610\;\mathrm{THz}$ and $n_{2}=1$ , the computed excitation momentum is $k_{1}=3.37\;\times \;10^{4}\;[1/m]$ . The K′ valley is excited within the Gap 2 frequency band, with excitation schematic illustrations for the armchair and zigzag edge states shown in Figure 10c and 10f, respectively. The resulting angles are calculated as follows:

(6) $$a_{4}=\arccos\frac{2.42\times 10^{4}}{3.37\times 10^{4}}=44.10{}^{\circ},\;a_{4}\prime =\arccos\frac{2.42\times 10^{4}}{2\times 3.37\times 10^{4}}=68.96{}^{\circ}$$

The computed results are consistent with Figure 9d,h.

Therefore, utilizing the momentum matching method offers a rigorous theoretical framework for explaining the phenomena of anomalous refraction and birefringence associated with the emission of light from dual-band valley-topological photonic crystals. It reveals the physical origin of the fixed angular relationship between the bifurcated output beams. Moreover, this approach enables accurate prediction of the outgoing beam angles, thus allowing precise control over beam routing. This capability has significant potential for applications in photonic computing and optical communication.

## 4. Liquid Crystal Tunable Edge States

**Figure 11:**
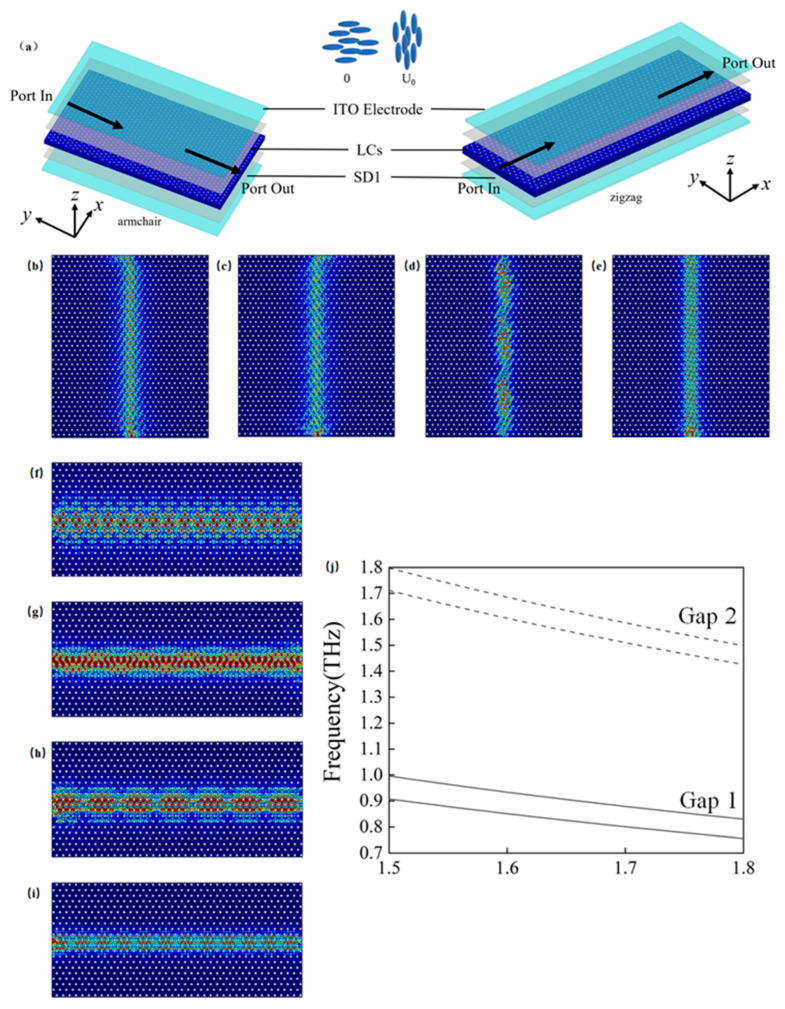
Electro-optic modulation of refractive index ( $n_{1}$ ) by NJU-LDn-4 and its effect on Gap 1 and Gap 2: (**a**) schematic of liquid crystal tunable armchair and zigzag edge states; (**b**) schematic for the armchair edge state at $n_{1}=1.5$ with an incident frequency of 0.996 THz; (**c**) schematic for the armchair edge state at $n_{1}=1.8$ with an incident frequency of 0.757 THz; (**d**) schematic for the armchair edge state at $n_{1}=1.5$ with an incident frequency of 1.798 THz; (**e**) schematic for the armchair edge state at $n_{1}=1.8$ with an incident frequency of 1.426 THz; (**f**) schematic for the zigzag edge state at $n_{1}=1.5$ with an incident frequency of 0.996 THz; (**g**) schematic for the zigzag edge state at $n_{1}=1.8$ with an incident frequency of 0.757 THz; (**h**) schematic for the zigzag edge state at $n_{1}=1.5$ with an incident frequency of 1.798 THz; (**i**) schematic for the zigzag edge state at $n_{1}=1.8$ with an incident frequency of 1.4260 THz; (**j**) schematic showing the changes in Gap 1 and Gap 2 under the influence of refractive index from 1.5 to 1.8.

The electro-optic effect of liquid crystals enables tunable transmission in dual-broadband terahertz photonic crystal edge states. The material NJU-LDn-4 [32,33,34,35,36] is used as the dielectric filling material (blue regions) described in Section 2.1. As depicted in Figure 11a, transparent indium tin oxide (ITO) electrodes are integrated on the top and bottom surfaces of the liquid crystal (LC) layer, allowing voltage application to modulate the refractive index of the LC. An alignment layer (SD1 [42]) controls the initial orientation of the LC molecules, with the director vector initially aligned horizontally along the x-direction. Upon applying a voltage of $U_{0}$ , the external electric field changes, resulting in the director vector of the LC aligning fully in the z-direction. Consequently, the refractive index $n_{1}$ can vary between 1.5 and 1.8. Under continuously varying voltage, Gap 1 and Gap 2 can shift between the frequency ranges of 0.908–0.996 THz and 0.757–0.830 THz, as well as between 1.711–1.798 THz and 1.420–1.492 THz. This results in dual-broadband transmissions of 2.40 THz and 3.72 THz, respectively. Figure 11b–e correspond to the armchair edge states at $n_{1\;}=1.5$ with a frequency of 0.996 THz; at $n_{1}=1.8$ with a frequency of 0.757 THz; at $n_{1}=1.5$ with a frequency of 1.798 THz; and at $n_{1}=1.8$ with a frequency of 1.426 THz, respectively. Figure 11f–i represent the zigzag edge states under the same refractive indices: $n_{1}=1.5$ at 0.996 THz; $n_{1}=1.8$ at 0.757 THz; $n_{1}=1.5$ at 1.798 THz; and $n_{1}=1.8$ at 1.426 THz. Figure 11j illustrates the computational results of refractive index $n_{1}$ on the band structures of Gap 1 and Gap 2.

By dynamically adjusting the refractive index of the LC, the transmission bands of guided waves can be dynamically tuned. This approach overcomes the inherent limitations of traditional waveguides, which are typically restricted to fixed frequency ranges after fabrication. Furthermore, the implementation of dual-band operation enables the optimization of waveguide performance based on practical requirements, thereby improving adaptability across diverse application scenarios.

## 5. Conclusions

To expand the practical applicability of valley-topological photonic crystal edge states and support future experimental realization, a liquid crystal tunable dual-band terahertz valley-topological photonic crystal is proposed. Frequency regions where edge states overlap with bulk states were identified and excluded, as they are unsuitable for valley edge state excitation. The resulting design supports excitation of the K valley (0.851–0.934 THz) and the K′ valley (1.604–1.686 THz).

Leveraging the momentum matching method, we further investigated the excitation conditions for anomalous refraction and birefringence. In the Gap 1 band, we achieved dynamic switching between anomalous refraction and birefringence through effective control of the exit environment. This technique not only enhances the control over light propagation characteristics but also opens new avenues for the design of novel optical devices.

To further improve the system’s tunability, we introduced the liquid crystal material NJU-LDn-4, which, under varying electric field, expanded the aforementioned dual bands to 0.757–0.996 THz and 1.426–1.798 THz. This tunable characteristic provides greater flexibility for related applications.

The findings presented herein offer significant potential for applications in tunable filter design, optical communication, photonic computing, optical sensing, and high-resolution imaging. By precisely controlling these edge states, we aim to provide an important theoretical foundation for the development of next-generation optical devices.

## Data Availability

The original contributions presented in the study are included in the article; further inquiries can be directed to the corresponding author.

## Supplementary Materials

The following supporting information can be downloaded at https://www.mdpi.com/article/10.3390/ma18122778/s1: Figure S1: Band structures of the unit cell at “θ = 30°” incidence. Left: PEC approximation for the metal. Right: Real material parameters (silver: ñ = 400 + i∙400; CS_2_: ñ = 1.6 + i∙0.01). The absence of significant differences confirms the validity of simplifying assumptions in the terahertz regime; Figure S2. The edge state configurations with realistic material parameters: armchair type (left) and zigzag type (right); Figure S3. Electric field distributions of edge states with realistic parameters: (a) armchair type at Gap 1 (0.880 THz); (b) armchair type at Gap 2 (1.632 THz); (c) armchair type at Gap 2 band gap (1.644 THz); (d) zigzag type at Gap 1 (0.880 THz); (e) zigzag type at Gap 2 (1.634 THz).

## Abbreviations

The following abbreviations are used in this manuscript:

MMI	Multimode Interference
MMF	Multimode Fiber
SMFs	Single-mode Fibers
SOAs	Semiconductor Optical Amplifiers
ITO	Indium Tin Oxide
LC	Liquid Crystal
