# Supplementary material for: Dual-Broadband Topological Photonic Crystal Edge State Based on Liquid Crystal Tunability
Source: Materials (Basel). 2025 Jun 12;18(12):2778. doi: 10.3390/ma18122778 (PMC12194887; doi:10.3390/ma18122778)
Supplement: Supplementary file 1 [file materials-18-02778-s001.zip › materials-3645168-supplementary.pdf]

Supplementary Materials

Dual-Broadband Topological Photonic Crystal Edge State Based on Liquid Crystal Tunability

Jinying Zhang [1,2,3,*,†], Bingnan Wang [1,†], Jiacheng Wang [1], Xinye Wang [1] and Yexiaotong Zhang [1]

[1] Beijing Key Lab for Precision Optoelectronic Measurement Instrument and Technology, School of Optics and Photonics, Beijing Institute of Technology, Beijing 100081, China
[2] Yangtze Delta Region Academy of Beijing Institute of Technology, Jiaxing 314001, China
[3] National Key Laboratory on Near-Surface Detection, Beijing 100081, China
* Correspondence: jyzhang@bit.edu.cn
† These authors contributed equally to this work.

Supplementary Note 1: The metallic scatterers in the THz band can be approximated as perfect electric conductors (PECs).

At a wavelength of 200 μm (corresponding to a frequency of 1.5 THz), the skin depth (δ) can be calculated as follows:

$$\delta = \sqrt{\frac{2}{\omega\mu\sigma}}$$

For a conductivity of $\sigma = 6.3 \times 10^7\ S/m$ and a permeability of $\mu = 4\pi \times 10^{-7} H/m,$ the calculated skin depth is approximately $\delta \approx 63nm$ at 1.5 THz (λ = 200 μm). The skin depth ($\delta \approx 63nm$) is significantly smaller than both the incident wavelength (200 μm) and the characteristic dimensions of the scatterer's structure.
According to Reference [36], the complex refractive index of silver at a wavelength of 248 nm can be expressed as: $\tilde{n} = 531 + i \cdot 689$
The reflectance R can be calculated as: $R = \left|\frac{1-\tilde{n}}{1+\tilde{n}}\right|^2 > 99.9\%$
In the terahertz regime, silver can be approximated as a perfect electric conductor (PEC) due to its exceptionally high reflectivity and negligible skin depth. To simplify numerical calculations across the entire THz frequency range, we adopt a constant complex refractive index of: $\tilde{n} = 400 + i \cdot 400$
For the dielectric component, carbon disulfide (CS_2) is chosen due to its well-characterized optical properties in the terahertz (THz) frequency range. According to Reference [37], its key parameters are:
Refractive index: $n \approx 1.62$ (nearly constant across the THz band)
Absorption coefficient: $\alpha < 6\text{cm}^{-1}$ (indicating low loss)
Based on the relation between the absorption coefficient α and the imaginary part of the refractive index k

$$k = \frac{\alpha\lambda}{4\pi}$$

where: $\lambda = 200\mu\text{m}$, substituting the values: $k < 10^{-2}$
By adopting a constant refractive index $\tilde{n} = 1.6 + i \cdot 0.01$, for CS_2, numerical simulations achieve a balance between computational efficiency and physical fidelity. The resulting band structure (Fig. S1) exhibits no discernible differences from the PEC-based reference case, validating the approximation for THz device design."

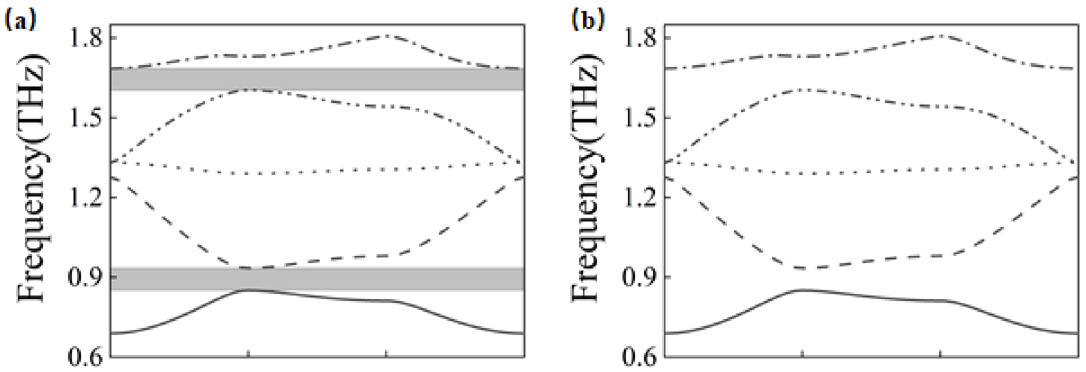

Figure S1. Band structures of the unit cell at θ = 30° incidence. Left: PEC approximation for the metal. Right: Real material parameters (silver: $\tilde{n} = 400 + i \cdot 400$; CS_2: $\tilde{n} = 1.6 + i \cdot 0.01$). The absence of significant differences confirms the validity of simplifying assumptions in the terahertz regime.

Supplementary Note 2: Transmission Calculation Methodology

When modeling the boundary-state structure shown in Fig. 3 with realistic material parameters (e.g., silver's large refractive index $\tilde{n} = 400 + i \cdot 400$), the finite-element method (FEM) requires excessive degrees of freedom (DoFs), exceeding the capacity of even a 256 GB RAM server. To enable feasible simulations while preserving physical accuracy, we eliminated parts of the boundary-layer structure that do not contribute to light transmission. The specific modeling is shown in Figure S2.

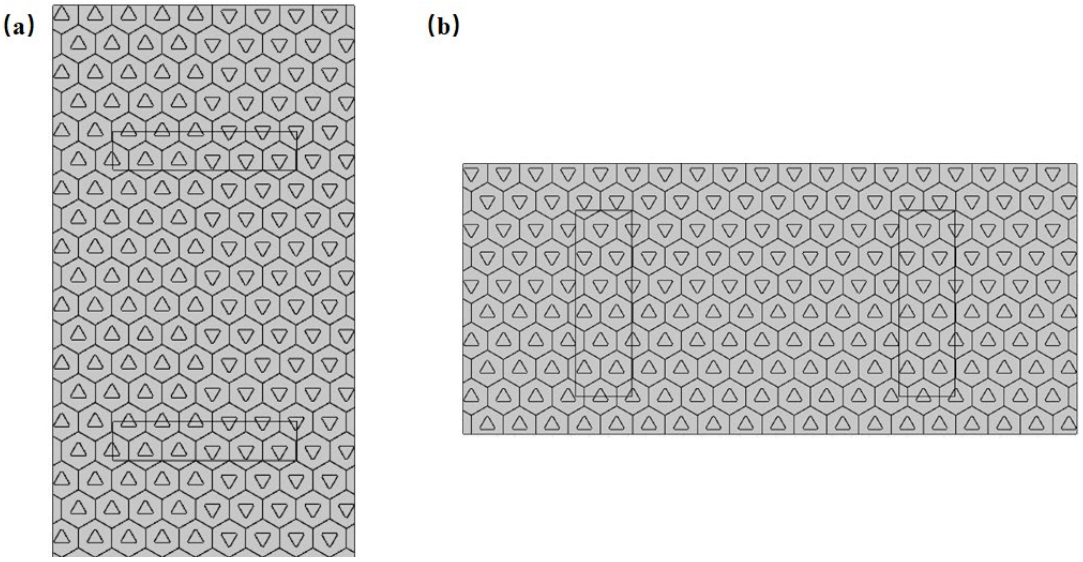

Figure S2. The edge state configurations with realistic material parameters: armchair type (left) and zigzag type (right).

The two rectangular frames correspond to electric field integration domains. To avoid energy localization at the input and output ports, these rectangular integration domains are strategically placed in the middle section of the boundary-state transmission channel, maintaining a separation of 10*a (where 'a' represents the lattice constant). To quantify the optical transmission through the boundary-state structure, we define the transmission ratio after propagation by 10 lattice constants (10*a) as:

$$T = \frac{|E_2|}{|E_1|}$$

where: $E_1 =$ Electric field amplitude at Integration Domain 1 (input reference); $E_2 =$ Electric field amplitude at Integration Domain 2 (10*a downstream). Figure S3 shows the electric field distribution, where it can be observed that the scatterers are no longer represented as blank spaces, yet light still cannot propagate through the metal scatterers Figure S3(c) shows the transmission profile of the armchair-type boundary state at 1.644 THz, where a bandgap appears as

depicted in Figure 2(d). The corresponding transmission rate is presented in Figure 4

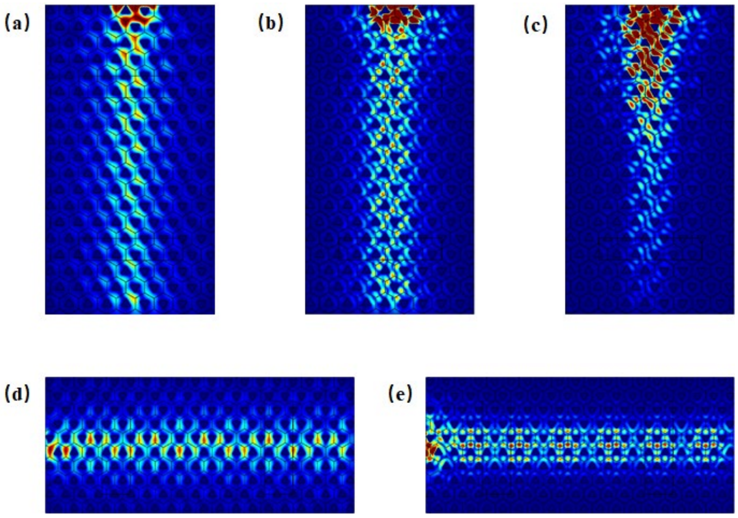

Figure S3. Electric field distributions of edge states with realistic parameters (a) armchair type at Gap 1（0.880THz） (b) armchair type at Gap 2（1.632THz）(c) armchair type at Gap 2 bandgap（1.644THz） (d) zigzag type at Gap 1（0.880THz）(e) zigzag type at Gap 2（1.634THz）.
